# Ketogenic Diet-induced Elevated Cholesterol, Elevated Liver Enzymes and Potential Non-alcoholic Fatty Liver Disease

**DOI:** 10.7759/cureus.6605

**Published:** 2020-01-08

**Authors:** Chika V Anekwe, Poongodi Chandrasekaran, Fatima C Stanford

**Affiliations:** 1 Weight Center, Massachusetts General Hospital, Boston, USA; 2 Internal Medicine, North Shore Physicians Group, Salem, USA; 3 Endocrinology and Pediatric Endocrinology, Massachusetts General Hospital, Boston, USA

**Keywords:** ketogenic diet, fatty liver, transaminitis, elevated liver enzymes, obesity, nutrition

## Abstract

A 57-year-old woman with class I obesity (BMI = 31.42 kg/m^2^) and a medical history significant for binge-eating disorder with emotionally-triggered eating, post-traumatic stress disorder, and untreated depression and anxiety, presented for follow-up of weight management with laboratory values revealing acutely-worsened hyperlipidemia and elevated liver enzymes. Abdominal ultrasound showed a mildly heterogenous and echogenic liver, without focal lesions, suggestive of non-alcoholic fatty liver disease. The only significant change from previous consultation four months prior was introduction of a ketogenic diet consisting of eggs, cheese, butter, oil, nuts, leafy green vegetables and milk (almond and coconut). The patient reported a reduction in hunger on this diet. Immediate discontinuation of the diet resulted in modest reduction of low-density lipoprotein cholesterol (LDL-C) and liver enzymes two weeks later. Resolution of liver enzymes was seen within eight months and LDL-C levels normalized one year later. This case report discusses the rationale, benefits and risks of a ketogenic diet and encourages increased vigilance and monitoring of patients on such a diet.

## Introduction

The ketogenic diet was originally developed for implementation under medical supervision to treat refractory epilepsy in infants and children [1]. It is a high-fat, low-carb, moderate-protein diet that produces metabolic changes similar to those seen in a state of starvation. These changes include increased levels of free fatty acids and serum ketones (acetoacetate, acetone and beta-hydroxybutyrate) and decreased levels of insulin, glucose and glucagon [2]. The theory is that ketone bodies are anti-convulsant when they cross the blood-brain barrier [3]. There are four types of ketogenic diets used for treating epilepsy - the classic ketogenic diet, the medium chain triglyceride diet, the modified Atkins diet and the low glycemic index treatment, each of which has respectively less restrictive requirements for fluid, protein and fat intake [4]. In recent years, however, the ketogenic diet has transitioned from a medically-monitored tool for treating epilepsy to become a mainstream interpretation of the low-carbohydrate dietary plan used to induce weight loss [2]. Individuals on ketogenic diets have been shown to lose and keep off more weight than those on low-fat diets. They also tend to report decreased hunger and maintain higher metabolism rates than low-fat dieters [5]. The ability to achieve and maintain weight reduction for individuals with overweight or obesity reduces cardiometabolic risk. Despite these benefits of the ketogenic diet, it is not completely without risk. In particular, it has the potential to increase blood cholesterol levels and induce elevations in liver enzymes. This case report illustrates the risks and benefits of the ketogenic diet.

## Case presentation

A 57-year-old woman with class I obesity, binge eating disorder, emotionally-triggered eating, post-traumatic stress disorder, depression and anxiety presented in 2012 with a BMI of 31.6 kg/m^2^ for treatment of her obesity. At initial evaluation, she reported no weight problems up until 2003, when she started noticing weight gain. At that point she was living in Iraq during the Iraq war, was very sedentary, stayed indoors most of the time and consumed a high-calorie diet. In 2008, she immigrated to the USA with her family. She continued to lead a sedentary lifestyle with rare formal exercise. She worked as the director of a social refugee agency and had many responsibilities caring for her household and family. She suffered from sleep disturbance and was taking clonazepam daily for sleep, which she obtained from her husband. She reported high stress levels, a strong desire to lose weight, and a lack of support in her daily life.

At initial presentation, she had symptoms consistent with dysthymia and was recommended to undergo treatment for mood stabilization with psychotherapy and/or psychopharmacology. She was also prescribed a low dose of topiramate, given off-label for appetite reduction. She suffered an adverse reaction to topiramate with an episode of significant anxiety and emotional outburst, resulting in a visit to the emergency department. Topiramate was discontinued and she began treatment with metformin for both obesity and metabolic syndrome; she was also instructed to introduce structured lifestyle changes including keeping records of dietary intake, exercise and sleep routine.

Metformin was not effective for weight reduction, and she in fact gained approximately 2.8 lbs during the four-month period during which the dose was titrated to 1000 mg twice daily. Although she was continued on metformin, she was recommended to discontinue using clonazepam for insomnia and instead start melatonin 3 mg and zonisamide 100 mg daily, both at bedtime. Zonisamide was titrated up to 200 mg at bedtime. Similar to topiramate, zonisamide is an anti-epileptic medication used off-label for appetite reduction in the treatment of obesity. As it can cause drowsiness, it is often dosed at bedtime. She lost 5.4 lbs (3% total body weight) within two months on this medication regimen, however was subsequently lost to follow-up, with her last visit on 5/21/13.

She was treated at an outside clinic from 2015 to 2018 with a variety of anti-obesity agents including naltrexone/bupropion, phentermine/topiramate ER and lorcaserin. Labs obtained on 2/24/16 showed hypercholesterolemia with total cholesterol (TC) = 271 mg/dL, low-density lipoprotein cholesterol (LDL-C) = 156 mg/dL and normal high-density lipoprotein cholesterol (HDL-C) = 102 mg/dL (see Table 1). Lipid values improved slightly with dietary modification and simvastatin, although she did not take simvastatin consistently. At her nadir weight in October 2015, she was 151 lbs (BMI = 28.5 kg/m^2^), and had achieved 17% reduction in total body weight from her heaviest weight of 182.8 lbs in March 2013.

**Table 1:** Patient's laboratory values before, during and after ketogenic diet KD: Ketogenic diet; AST: Aspartate aminotransferase; ALT: Alanine aminotransferase; LDL-C: Low-density lipoprotein cholesterol; Tot-C: Total cholesterol; HDL-C: High-density lipoprotein cholesterol; TG: Triglycerides.

Laboratory reference ranges	Prior to KD	During KD	Two weeks after KD	Eight months after KD	One year after KD
AST (15-41 U/L)	21 U/L	67 U/L	55 U/L	27 U/L	-
ALT (10-35 U/L)	18 U/L	119 U/L	80 U/L	25 U/L	-
LDL-C (40-130 mg/dL)	156 mg/dL	216 mg/dL	209 mg/dL	157 mg/dL	80 mg/dL
Tot-C (0-200 mg/dL)	271 mg/dL	323 mg/dL	-	268 mg/dL	-
HDL-C (>39 mg/dL)	102 mg/dL	98 mg/dL	-	84 mg/dL	-
TG (0-150 mg/dL)	66 mg/dL	45 mg/dL	-	133 mg/dL	-

The patient returned for follow-up of obesity management in April 2018. At this point she was off all anti-obesity medications and was in fact on the weight-promoting medication seroquel; at 170.5 lbs, she had regained a significant portion of her lost weight. She was restarted on bupropion and zonisamide. In September 2018, the patient self-initiated a ketogenic diet, consuming predominantly eggs, cheese, butter, oil, nuts, leafy green vegetables and almond/coconut milk. This resulted in a modest weight loss of about 6 lbs over two months. However, she also suffered a marked increase in liver enzymes and total and LDL cholesterol levels.

**Figure 1:**
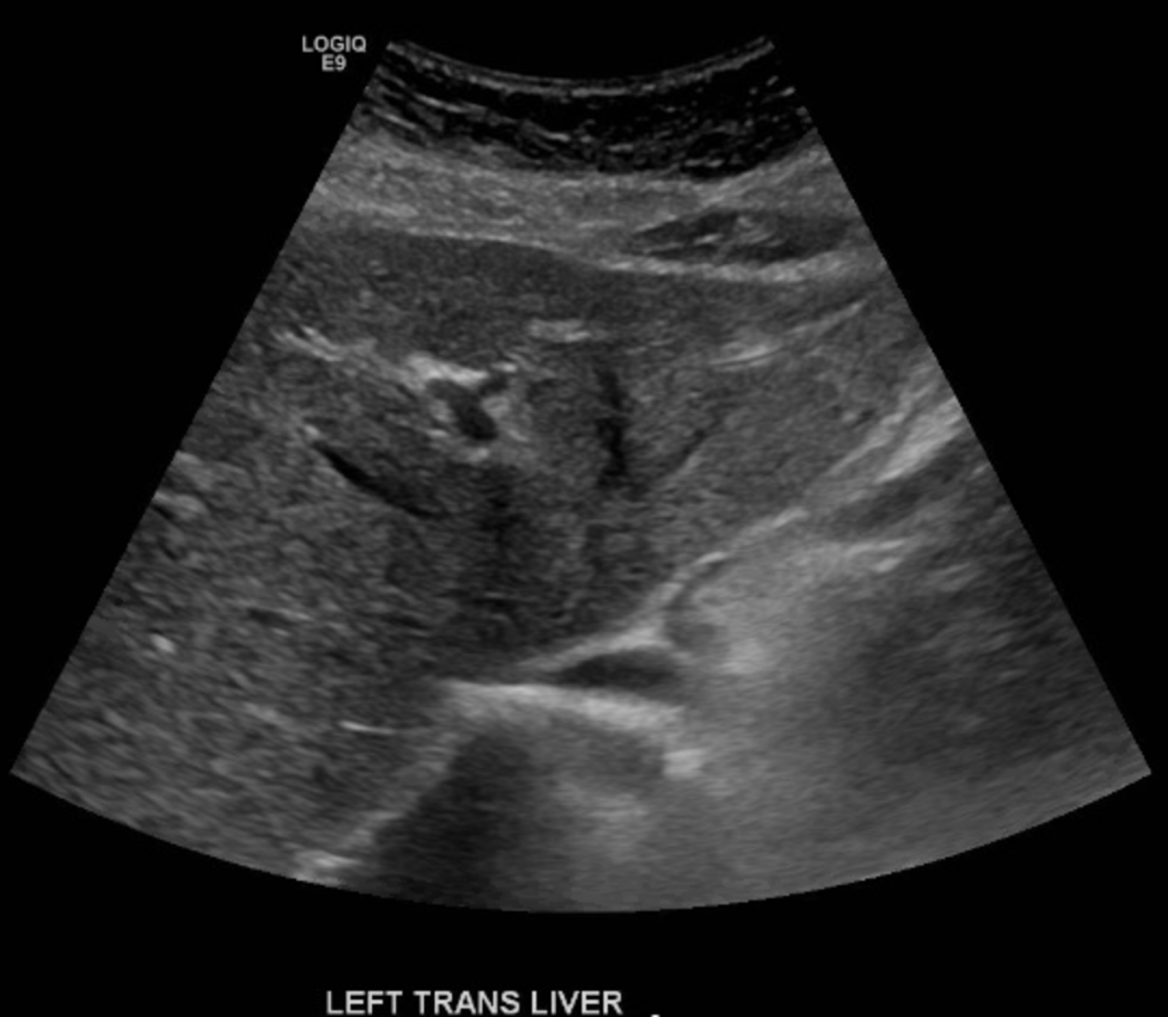
Left transverse view of liver

**Figure 2:**
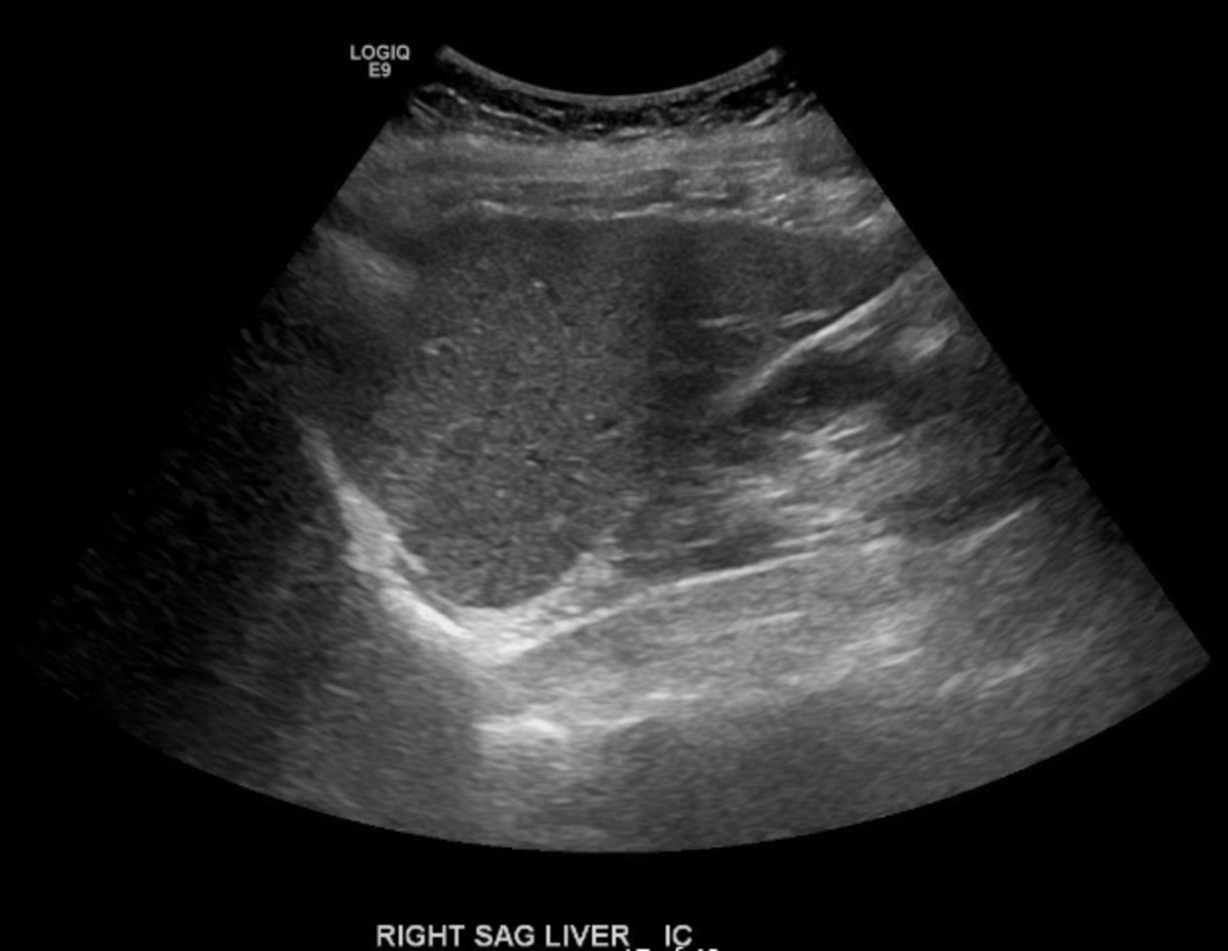
Right sagittal view of liver

Laboratory testing on 12/21/18 revealed aspartate aminotransferase (AST) = 67 U/L and alanine aminotransferase (ALT) = 119 U/L (alkaline phosphatase was normal at 77 U/L). TC = 323 mg/dL and LDL-C = 216 mg/dL (triglycerides, TG, were normal at 45 mg/dL). Also of note was an elevated Vitamin B12 level of 1,156 pg/mL, despite the patient not taking any B12 supplementation. In addition, 25-hydroxy Vitamin D levels were insufficient, at 22 ng/mL, and ferritin levels were elevated at 155 ug/L. Previous TC level obtained by her primary physician on 3/15/18 was 267 mg/dL; LDL-C and TG values were not obtained. Previous liver chemistries on 4/10/18 were within normal limits, with AST = 21 U/L and ALT = 18 U/L (see Table 1). Abdominal ultrasound obtained on 1/10/19 revealed a mildly heterogenous and echogenic liver, with no focal lesions visualized and no significant biliary ductal dilation (see Figure 1 and Figure 2). These findings are highly suggestive of hepatic steatosis, or fatty liver disease.

The patient agreed to discontinue the ketogenic diet and follow up with a registered dietician. She continued bupropion 150 mg twice daily and zonisamide 200 mg in the evening. She also continued cholecalciferol 2000 IU daily for hypovitaminosis D. She was encouraged to consume a high-quality diet and engage in regular physical activity. In addition, due to her LDL-C value of 216, she was prescribed atorvastatin 20 mg daily. The National Cholesterol Education Program Adult Treatment Panel III recommends statin therapy for low-risk individuals (one or no risk factors) who have an LDL-C > 190 mg/dL, with lower LDL-C cut-off values for higher risk populations [6]. The patient has no reported history of premature cardiovascular events in first-degree relatives; LDL-C levels of her first-degree relatives were not accessible.

The patient followed up with her primary physician four days after her weight management visit and reported having stopped the ketogenic diet, while affirming adherence to a high-quality diet and regular exercise. She also reported taking an omega-3 DHA/EPA 1000 mg (120 mg/180 mg) fish oil capsule daily. Her weight was 164 lbs. She received counseling to follow a low-fat and low-carbohydrate diet rich in fruits and vegetables. She was counseled to engage in routine aerobic exercise at least three times per week and advised against implementing any diet that promotes rapid weight loss. Repeat laboratory testing 10 days after visit to the primary physician revealed improved liver enzymes (AST = 55 U/L, ALT = 80 U/L), and a slightly decreased direct LDL-C of 209 mg/dL. Liver enzymes resolved completely within eight months, while LDL-C levels resolved by one year (see Table 1). She was advised to continue follow-up for monitoring of weight and laboratory values as well as continued lifestyle counseling.

## Discussion

Individuals with obesity or overweight often implement what they hope to be the next “quick fix” for reversing their increased fat mass. Often these self-initiated diets are implemented without the guidance of a licensed health care provider. The ketogenic diet is one example of a dietary pattern that has gained popularity, with mainstream use as an effective strategy for weight loss.

The ketogenic diet was originated in the 1920s and 1930s as an alternative to fasting for the reduction of seizure frequency in children with epilepsy [1]. Individuals in ketosis release ketone bodies from the breakdown of body fat, and these ketones are used, instead of glucose, as the primary source of energy [2]. This ketotic state has been shown to alter genes involved in energy metabolism in the brain, which helps stabilize the function of neurons susceptible to epileptic seizures [3].

The ketogenic diet is very low in carbohydrates and very high in fat. Clinical ketogenic diets limit carbs to 20 to 50 g per day, primarily from non-starchy vegetables, with very low carb ketogenic diets restricting carbs to less than 20 g per day [5]. Protein is kept high enough to maintain lean body mass, but low enough to preserve ketosis. The amino acids alanine and glutamine can be converted to glucose through gluconeogenesis, thus removing the body from a ketotic state [7].

The diet works, simply, by altering energy metabolism. After three to four days of fasting or following a very low-carbohydrate diet, the body becomes deprived of dietary sugar and starch, and reacts by reducing insulin secretion and switching to primarily burning fat for fuel. The resulting overproduction of acetyl-CoA leads to formation of ketones (acetoacetate, acetone and beta-hydroxybutyric acid) in a process known as ketogenesis [8]. While the brain is unable to use fatty acids for fuel, ketones can cross the blood-brain barrier, thereby providing fuel to the typically glucose-dependent brain. The full transition to physiological, or nutritional, ketosis usually takes a week [8]. The true ketogenic diet contains 75% to 90% calories from fat, 10% from protein, and 5% from carbs. Careful monitoring of dietary intake and blood (not urinary) ketone levels is required in order to ensure an adequate state of ketosis. Protein intake may need to be increased for individuals doing resistance training, in order to prevent muscle degradation [2].

The ketogenic diet has both benefits and risks. Advantages of the diet include weight loss, reduction in cravings and appetite (likely due to the satiating effects of fat and protein as well as the stabilizing effect on blood sugar levels), and a more stable flow of energy to organs and tissues, due to the reliance of fat catabolism rather than dietary intake for energy [2, 7]. The weight loss occurs partly due to the diuretic effect of glycogen utilization and the likely calorie reduction resulting from the restricted dietary variety, but primarily because the reduction in blood glucose and insulin leads to less fat storage, as insulin is known to promote the conversion of excess glucose to fat [5]. Research also suggests that the ketogenic diet improves insulin sensitivity and glycemic control, although the mechanisms are unclear [8].

One potential risk of the ketogenic diet is an increase in LDL-C, TC and liver enzymes. Notably, in rodents, development of nonalcoholic fatty liver disease (NAFLD) and insulin resistance have been described [9]. Despite this risk, some studies show that the higher-risk small dense LDL particles were decreased in individuals on a ketogenic diet, while HDL cholesterol and triglycerides tend to improve [9, 10]. It should be noted, however, that the reduction in small dense LDL particles is observed only in individuals with certain variants of the apolipoprotein gene which is known to play a key role in lipid metabolism [11]. Depending on an individual’s response to the diet, benefits of improved glycemic control may outweigh potential risks of an elevated LDL. One way to mitigate the negative effects of the diet on LDL cholesterol is to replace saturated fats from animal sources with polyunsaturated fats found in avocados, nuts, seeds, coconut and olive oil.

Another side effect of the ketogenic diet is a constellation of symptoms known as “keto flu,” which includes lightheadedness, fatigue, headaches, nausea, and constipation. These symptoms are a result of the body’s rapid excretion of sodium and fluids as carbohydrate intake is restricted and glycogen stores are depleted. Increasing sodium by 1-2 g per day may restore electrolyte balance [2].

Finally, the extreme limitation of carbohydrates in a ketogenic diet poses concern regarding the potential impact on micronutrient intake and gut health. Ketogenic diets eliminate not only sugar and refined carbohydrates but also pulses, whole grains, fruits and starchy vegetables, all of which contain vitamins, minerals, antioxidants, phytochemicals and fiber, including healthy gut microbiota-promoting prebiotic fiber. Although this alteration in the gut microbiome may be beneficial for individuals with epilepsy, research is lacking on the impact on populations using the diet for weight loss or diabetes management [8].

## Conclusions

The ketogenic diet is a high-fat, moderate-protein, low-carbohydrate diet that can induce weight loss and improvement in glycemic control, but poses a risk of inducing hyperlipidemia, elevation of liver enzymes and onset of fatty liver disease. Like any other restrictive dietary plan, the ketogenic diet is often difficult to maintain long-term. Cycling in and out of ketosis reduces its metabolic effects. Patients on a ketogenic diet should be monitored with frequent laboratory testing of blood ketones, lipids, and liver enzymes as well as frequent assessment of cognitive function and energy levels.

## References

[1] Kossoff EH (2019). Ketogenic dietary therapies for the treatment of epilepsy. UpToDate.

[2] Dennett C (2019). The ketogenic diet for weight loss. Today's Dietitian.

[3] Emory University Health Sciences Center (2005). Ketogenic diet prevents seizures by enhancing brain energy production, increasing neuron stability. Science Daily.

[4] Sampaio LP (2016). Ketogenic diet for epilepsy treatment. Arq Neuropsiquiatr.

[5] Abbasi J (2018). Interest in the ketogenic diet grows for weight loss and type 2 diabetes. JAMA.

[6] Last AR, Ference JD, Falleroni J (2011). Pharmacologic treatment of hyperlipidemia. Am Fam Physician.

[7] Brouns F (2018). Overweight and diabetes prevention: is a low-carbohydrate — high-fat diet recommendable?. Eur J Nutr.

[8] Paoli A (2014). Ketogenic diet for obesity: friend or foe?. Int J Environ Res Public Health.

[9] Kosinski C, Jornayvaz FR (2017). Effects of ketogenic diets on cardiovascular risk factors: evidence from animal and human studies. Nutrients.

[10] Volek JS, Sharman MJ, Forsythe CE (2005). Modification of lipoproteins by very low-carbohydrate diets. J Nutr.

[11] Ordovas JM (1999). The genetics of serum lipid responsiveness to dietary interventions. Proc Nutr Soc.

